# Effectiveness and Tolerability of Topical Amitriptyline 10% Plus Lidocaine 2% Gel in Adults With Post‐Traumatic Trigeminal Neuropathic Pain: A Real‐World Evidence Study

**DOI:** 10.1111/joor.70209

**Published:** 2026-05-05

**Authors:** Ashley Lebel, Imen Stambouli, Yves Boucher

**Affiliations:** ^1^ Department of Orofacial Pain Institute of Dental Surgery, Pitié‐Salpêtrière Hospital (AP‐HP) Paris France; ^2^ Laboratory of Orofacial Neurobiology Inserm UMR1333 Oral Heath, Université Paris Cité Montrouge France; ^3^ Gene Regulation and Adaptive Behaviors Center for Neuroscience Sorbonne University (NeuroSU), Sorbone Université Paris France; ^4^ Unit of Orofacial Pain and Temporomandibular Dysfunction University Dental Clinic Monastir Tunisia

**Keywords:** dental implants, endodontics, painful post‐traumatic trigeminal neuropathy, tooth extraction, topical analgesic, trigeminal nerve injuries, trigeminal neuralgia

## Abstract

**Background:**

Post‐traumatic trigeminal neuropathic pain (PTNP) is a debilitating orofacial pain disorder most commonly caused by trigeminal nerve injury after dental procedures or facial trauma. Available systemic therapies have modest and inconsistent efficacy, with substantial tolerability concerns limiting adherence.

**Objectives:**

To evaluate the real‐world effectiveness and tolerability of a compounded intraoral mucoadhesive gel containing amitriptyline 10% plus lidocaine 2% in adults with PTNP.

**Methods:**

This retrospective real‐world evidence study included adults with PTNP meeting ICHD‐3/ICOP criteria who were treated at a tertiary orofacial pain clinic from May 1, 2023, to April 30, 2024. The primary outcome was the within‐patient change in pain intensity on a 0–10 numerical rating scale (NRS) from baseline to week 8. Secondary outcomes included responder rates (≥ 50% and ≥ 30% pain reduction), patient global impression of improvement (PGI‐I) and adverse events (AEs).

**Results:**

Of 94 patients identified, 40 were included in the complete‐case initiator cohort. Mean pain intensity decreased from 6.3 ± 1.7 at baseline to 3.0 ± 2.7 at week 8, corresponding to a mean within‐patient reduction of 3.30 points (95% CI, 2.51–4.09; *p* < 0.001). Twenty‐two of 40 patients (55%; 95% CI, 40–69) achieved a ≥ 50% reduction in pain intensity, and 27 of 40 (68%; 95% CI, 52–80) achieved a ≥ 30% pain reduction. Seventy‐five percent (30/40, 95% CI, 60–86) reported overall improvement on the PGI‐I. Mild treatment‐related AEs occurred in five of 40 patients (12%); no serious AEs or treatment discontinuations were reported.

**Conclusion:**

In this retrospective real‐world study, topical amitriptyline–lidocaine mucoadhesive gel was associated with statistically significant and clinically meaningful pain improvement in adults with PTNP, together with favourable tolerability. These findings support targeted mucosal delivery as a promising local therapeutic option and potential medication‐sparing strategy, particularly when systemic therapies are ineffective or poorly tolerated. Randomized controlled trials are now needed to confirm efficacy and clarify the role of topical amitriptyline in PTNP treatment pathways and routine clinical care.

## Graphical abstract

A compounded amitriptyline 10% plus lidocaine 2% mucoadhesive gel was associated with substantial pain improvement and favourable tolerability in adults with post‐traumatic trigeminal neuropathic pain (PTNP). These findings support targeted mucosal delivery as a promising practical local option and a potential medication‐sparing strategy, particularly when systemic therapies are ineffective or poorly tolerated.

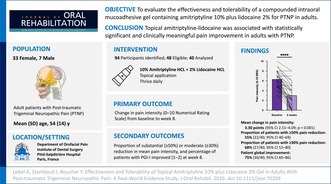

## 1 Background

Post‐traumatic trigeminal neuropathic pain (PTNP), classified as “painful post‐traumatic trigeminal neuropathy” in the International Classification of Headache Disorders, 3rd edition (ICHD‐3) [1] and “post‐traumatic trigeminal neuropathic pain” in the International Classification of Orofacial Pain (ICOP) [2], is a debilitating neuropathic pain disorder resulting from injury to branches of the trigeminal nerve that commonly occurs after dental and maxillofacial procedures. Prevalence estimates vary widely by clinical setting, procedure, and follow‐up, ranging from 1.6% to 13% following third‐molar extraction, local anaesthesia, dental implant placement, and root canal treatment [3, 4, 5].

Patients typically present with spontaneous intraoral and facial burning or shooting pain, dysesthesia, and stimulus‐evoked allodynia, accompanied by concurrent positive and negative sensory signs [1, 2, 3]. Nonetheless, PTNP remains underrecognized among dental and pain clinicians, contributing to diagnostic delays and misattribution to dentoalveolar pathology, thereby prolonging patient suffering and often leading to unnecessary iatrogenic interventions [4, 6, 7]. PTNP imposes a substantial individual and public‐health burden, adversely affecting quality of life [8], psychosocial and affective functioning [9], and health‐care utilization with considerable economic costs to patients and health systems [10].

Current pharmacologic management of PTNP is largely extrapolated from NeuPSIG neuropathic pain guidelines, which usually recommend tricyclic antidepressants (TCAs), serotonin–noradrenaline reuptake inhibitors (SNRIs) and gabapentinoids (α2δ‐ligands) as first‐line treatments [11]. However, average efficacy is modest, and systemic adverse events commonly limit dosing and adherence in routine practice, with low‐certainty evidence for oral amitriptyline specifically [12]. Real‐world outcomes are disappointing, with approximately 89% of PTNP patients showing inadequate response to stepped systemic pharmacotherapy [13].

These limitations have generated growing interest in topical therapies that target peripheral nociceptors to reduce abnormal input from injured nerve afferents while minimizing systemic exposure. Comprehensive reviews of topical analgesics and neuropathic pain pathophysiology support the mechanistic and clinical rationale for local delivery [14, 15, 16, 17, 18], particularly in the oral cavity, where the mucosa's high permeability and dense trigeminal innervation may be therapeutically leveraged [19, 20, 21].

Amitriptyline (AMT), usually administered orally, also exerts potent local actions aligned with PTNP pathophysiology, including blockade of voltage‐gated sodium channels (Na_v_1.7/1.8/1.9) and modulation of TRP‐channel (TRPA1, TRPV1 at higher concentrations) pathways implicated in peripheral nerve injury‐driven sensitization [22, 23, 24]. Translational and early‐phase clinical studies, together with systematic reviews, suggest that high‐concentration topical AMT formulations may provide analgesic and antinociceptive effects in peripheral neuropathic pain with favourable tolerability [25, 26, 27, 28]. Notably, intraoral tricyclic formulations at low concentrations have demonstrated significant, long‐lasting pain reduction and excellent tolerability in healthy volunteers, radiochemotherapy‐induced oral pain, and burning mouth syndrome (BMS), supported by moderate [29, 30, 31] and high‐quality evidence [32, 33, 34, 35, 36, 37, 38, 39]. Although BMS and PTNP differ nosologically, both share peripheral trigeminal small‐fibre dysfunction and overlapping nociceptive mechanisms [40, 41, 42], providing biological justification for therapeutic extrapolation.

Despite a strong mechanistic rationale and supportive evidence in related conditions, topical AMT has not been systematically evaluated in PTNP. Addressing this evidence gap is particularly relevant given the substantial unmet clinical need in this population.

We therefore conducted a real‐world, retrospective observational study to evaluate the effectiveness, safety, and tolerability of intraoral topical amitriptyline 10% plus lidocaine 2% gel in adults with PTNP. We hypothesized that this intervention would be associated with clinically meaningful pain reduction, favourable responder rates and minimal adverse events.

## 2 Methods

### 2.1 Design and Setting

This real‐world, monocentric, observational study included adults with PTNP treated with a compounded intraoral amitriptyline 10% plus lidocaine 2% mucoadhesive gel at the Department of Orofacial Pain (DOFP) of the Pitié‐Salpêtrière Hospital in Paris, France, between May 1, 2023, and April 30, 2024. This study followed the Initiative on Methods, Measurement, and Pain Assessment in Clinical Trials (IMMPACT) recommendations and the STrengthening the Reporting of OBservational studies in Epidemiology (STROBE) guidelines. The complete‐case initiator cohort comprised 40 patients with baseline and week 8 NRS assessments. No a priori statistical power calculation was conducted; sample size was determined by the available eligible patient population during the study period, providing preliminary precision for effect size estimation to inform future trials. We summarized patient flow and analysis denominators in a STROBE‐style diagram (Figure S1).

### 2.2 Ethics

The study complied with the Declaration of Helsinki and French data‐protection regulations (National Data Protection Agency registration #DR‐2020‐341). The Assistance Publique‐Hôpitaux de Paris Institutional Review Board approved this study (INDS‐TPS#1106180) and waived informed consent owing to the use of retrospective, de‐identified data collected during routine clinical care.

### 2.3 Participants

The DOFP is a tertiary clinical setting receiving inpatient and outpatient referrals from dentists, oral and maxillofacial surgeons, primary care physicians, specialist physicians, and emergency departments. We retrospectively screened consecutive charts from electronic medical records (EMRs) and selected patients with a principal diagnosis of PTNP according to ICHD‐3/ICOP. Diagnoses were made by a senior orofacial pain DDS and independently verified by two blinded senior DDS specialists using a standardized ICHD‐3/ICOP checklist to confirm diagnostic reproducibility and eligibility. We then identified those prescribed topical amitriptyline gel with at least one in‐person 8‐week follow‐up visit. Eligibility was assessed at baseline; post‐baseline deviations did not affect eligibility and were addressed in prespecified sensitivity analyses. Baseline demographic and clinical characteristics were summarized using descriptive statistics as described below. Patients who received a prescription but did not initiate the gel were not included in the primary analysis and are shown in Figure S1 with reasons for non‐initiation.

Inclusion criteria were: (1) PTNP per ICOP/ICHD‐3; (2) prescription of a topical intraoral gel containing amitriptyline 10% plus lidocaine 2%; (3) treatment‐naïve for topical amitriptyline or stable systemic or topical neuropathic analgesic regimens, defined as no initiation or change within 4 weeks preceding baseline; and (4) no restrictions based on age, sex, gender, or ethnicity.

Exclusion criteria included: (1) not meeting ICHD‐3/ICOP criteria for PTNP; (2) active dentoalveolar pathology at the painful site that could account for pain; (3) known hypersensitivity or contraindications to amitriptyline or lidocaine, including amide local anaesthetics; (4) concurrent topical analgesics applied to the target site that were intended to be continued; (5) non‐standard topical formulation or concentration at baseline; (6) conditions preventing valid self‐report (such as severe cognitive impairment) or safe intraoral application (such as erosive mucosal conditions); and (7) pregnancy or breastfeeding, per institutional policy.

### 2.4 Topical Mucoadhesive Formulation Preparation

Topical amitriptyline hydrochloride 10% with lidocaine hydrochloride 2% was compounded extemporaneously as individualized, named‐patient magistral preparations, each prepared on a unique prescription, in a single registered community pharmacy (French officine), in accordance with ANSM (National Agency for Medicines and Health Products Safety) Good Preparation Practices. Application and local dosing instructions were determined by clinical judgement and previously published protocols [23, 24]. The formulation consisted of amitriptyline hydrochloride (5 g; final concentration 10%) and lidocaine hydrochloride (1 g; final concentration 2%), both pharmaceutical‐grade raw materials complying with current European Pharmacopoeia monographs, incorporated into NovaFilm Gel Base (MEDISCA), a hypoallergenic, mucoadhesive aqueous gel base free of methylisothiazolinone and methylchloroisothiazolinone (MI/MCI), parabens, petrolatum, mineral oil and alcohol, to a final volume of 50 mL. No additional cosolvent or penetration enhancer was added. The gel was dispensed in a 50‐mL airless pump bottle delivering 0.25 mL per actuation.

For compounding, the amitriptyline hydrochloride and lidocaine hydrochloride powders were first weighed and triturated together in a clean, dry mortar and pestle to obtain a fine, homogeneous powder mixture. The NovaFilm gel base was then incorporated progressively by geometric dilution, with trituration between each addition until a visually homogeneous gel was obtained. The preparation was transferred into the 50‐mL airless pump container (Medisca, Saint‐Laurent, Québec), labelled, and dispensed after routine checks of appearance, homogeneity and correct pump dispensing. The beyond‐use date assigned by the pharmacy was 2 months from the date of preparation; formulations were stored in their original container at room temperature and protected from light.

Patients were instructed to apply one pump actuation (0.25 mL; approximately 25 mg amitriptyline hydrochloride), corresponding to a pea‐sized amount, to the symptomatic mucosal site, most often the painful gingival or adjacent alveolar/buccal mucosa, three times daily using a standardized application technique designed to preferentially promote local mucosal exposure while minimizing inadvertent swallowing; no intraoral splint or neurosensory stent was used. Before each application, the target mucosal area was briefly dried by blotting with gauze or tissue. The gel was then applied and held in place for 2 min, consistent with previously published topical buccal contact‐time protocols [29, 32, 35]. Immediately thereafter, any visible nonadherent excess was removed by gentle wiping, followed by a brief water rinse with expectoration. Patients were instructed to spit residual saliva for several minutes, refrain from vigorous swishing and avoid eating or drinking for 15 min after each application.

### 2.5 Outcomes and Measures

The primary outcome was the change in pain intensity on a 0–10 NRS from baseline to week 8. Secondary outcomes included the proportion of patients achieving either a substantial (≥ 50%) or moderate (≥ 30%) reduction in mean pain intensity from baseline, and the percentage of patients reporting improvement on the PGI‐I (score 1–2) at week 8. Additional outcome measures included adherence to treatment at week 8 and the incidence of TEAEs, SAEs, and AEs leading to treatment discontinuation.

#### 2.5.1 Pain Assessment

Pain intensity was assessed using a 0–10 NRS daily pain diary at baseline (the mean of the last 3 days before the treatment period) and after treatment (the mean of the last 3 days of the treatment period) to reduce recall bias. Neuropathic characteristics were appraised according to the NeuPSIG grading system [43], and using the Douleur Neuropathique 4 (DN4), a questionnaire designed to screen for neuropathic pain components (0–10; screening cutoff ≥ 4). Pain duration was defined as the time between the onset of pain and the consultation date. Pain location was evaluated with a map of the face, mouth, and oral mucosa (e.g., lips, palate), and the affected trigeminal division was identified according to the injury‐related area. A qualitative bedside sensory examination of the injured and contralateral areas was also performed, as described elsewhere [44], to evaluate sensitivity to touch, cold and pinprick stimuli. Response to treatment was defined as substantial (≥ 50%), moderate (30%–49%), or minimal (15%–29%) reduction in pain intensity from baseline [45].

#### 2.5.2 Emotional Functioning

Patients completed the Hospital Anxiety and Depression Scale (HADS), including the HADS‐Anxiety (HADS‐A) and HADS‐Depression (HADS‐D) subscales.

#### 2.5.3 Global Improvement

Global improvement was evaluated using the Patient Global Impression of Improvement (PGI‐I) 7‐point scale (1 = “very much improved”, 2 = “much improved”, 3 = “a little improved”, 4 = “no change”, 5 = “a little worse”, 6 = “much worse”, 7 = “very much worse”); improvement was defined as a PGI‐I score of 1 or 2.

#### 2.5.4 Adherence

Adherence was defined a priori and dichotomized as adherent versus nonadherent based on chart documentation of self‐reported ongoing use at the 8‐week visit and ≥ 2 applications/day on ≥ 5 of the last 7 days, corroborated by dispensing records when available.

#### 2.5.5 Safety and Tolerability

Treatment‐emergent adverse events (TEAEs), serious adverse events (SAEs), and adverse events (AEs) leading to treatment discontinuation were reported and graded using the Common Terminology Criteria for Adverse Events (CTCAE) v5.0. Patients were monitored for local AEs (e.g., mucosal irritation, taste alteration, numbness) and systemic AEs, especially for systemic TCA‐related AEs (e.g., sedation, dry mouth, somnolence, dizziness, palpitations). Clinical observations that did not meet AE criteria were also noted for context.

### 2.6 Statistical Analysis

The primary analysis used a complete‐case initiator cohort, including patients who initiated the prescribed intraoral gel, defined as documented pharmacy dispensing or chart‐confirmed start, and had both baseline and week 8 NRS assessments (week 8 window, day 56 ± 7). Patients who received a prescription but had no evidence of treatment initiation were included in the flow diagram and descriptive counts but were excluded from the primary analysis. Reasons for non‐initiation were abstracted when available. Other variables were summarized on an available‐case basis, with denominators reported.

Baseline continuous variables were summarized using mean ± SD or median (IQR), as appropriate, and categorical variables were summarized as n/N (%). The primary endpoint was the within‐patient change in NRS pain intensity from baseline to week 8. Because the estimand of interest was the mean within‐patient change in pain intensity, changes from baseline were assessed using a paired *t*‐test and reported as mean change with 95% CIs. Given the bounded and ordinal nature of the NRS and the non‐normal distribution of paired change scores, a Wilcoxon signed‐rank sensitivity analysis was performed to assess robustness. Supportive nonparametric summaries included the Hodges–Lehmann pseudo‐median paired difference with 95% CIs.

Responder proportions for at least 30% and at least 50% pain reduction, PGI‐I improvement, and adverse‐event proportions were reported with Wilson score 95% CIs. Five prespecified sensitivity analyses were conducted: (i) per‐protocol analysis restricted to participants adherent at week 8 follow‐up, (ii) exclusion of participants with initiation or dose change of systemic analgesic during follow‐up, (iii) exclusion of participants receiving concurrent systemic tricyclics at baseline, (iv) stratification by baseline pain severity (NRS < 6 vs. ≥ 6) to assess potential baseline‐severity effects, and (v) restriction to participants with pain duration of at least 3 months and at least 6 months at baseline to mitigate bias from spontaneous early natural recovery. An additional post hoc analysis examined results stratified by pain duration of less than 12 months versus 12 months or longer at baseline.

A two‐tailed *p* < 0.05 was considered statistically significant. Given the exploratory nature of this retrospective real‐world evidence study, no formal adjustment for multiple testing was applied to secondary outcomes. Interpretation emphasized effect estimates, confidence intervals, and clinical relevance rather than *p*‐values alone. Unless otherwise specified, CIs are 95%, exact *p*‐values are reported to three significant digits with very small values presented as *p* < 0.001, and denominators reflect available‐case data. All analyses were conducted from May 2024 to February 2026 using Stata/MP 17.0 (StataCorp LLC, College Station, TX, USA) and GraphPad Prism 10.2.1 (GraphPad Software, San Diego, CA, USA).

## 3 Results

### 3.1 Participants

Of 94 patients with PTNP identified, 48 met baseline eligibility. The primary complete‐case cohort comprised 40 patients who initiated treatment and had both baseline and week 8 NRS assessments. Table 1 and Table 2 summarize baseline characteristics. Figure S1 shows the source cohort flow, the complete‐case primary analysis set, and sensitivity analysis subsets with reasons documented. Mean age was 54 ± 14 years; 33/40 (83%) were female. Among female patients, 23/33 (70%) were postmenopausal, 2/33 (6%) were perimenopausal, and 8/33 (24%) were premenopausal. Baseline HADS‐A was 9 (IQR 7–11) and HADS‐D 5 (IQR 3–7); HADS was available for 35/40 patients, of whom 22/35 (63%) and 11/35 (31%) had HADS‐A ≥ 8 and HADS‐D ≥ 8, respectively. Baseline DN4 was 3 (IQR 2–5); 16/40 patients (40%) scored ≥ 4. Tooth extraction was the most common initiating event (14/40 [35%]); extraction sites were third molars in 6/14 (43%), first or second molars in 6/14 (43%), and premolars in 2/14 (14%). Other aetiologies included root canal treatment in 13/40 (33%), dental implant placement in 5/40 (13%), periodontal surgery in 4/40 (10%), head trauma in 2/40 (5%) and maxillofacial surgery in 2/40 (5%). Median pain duration, defined as the time from injury to treatment initiation, was 24 months (IQR 11–48), with no evidence of differences across aetiological categories (Kruskal–Wallis H = 1.067; *p* = 0.957) (Table S2). Pain distribution was maxillary in 18/40 (45%), mandibular in 20/40 (50%), and both maxillary and mandibular in 2/40 (5%); pain was unilateral in 35/40 (88%) unilateral and bilateral in 5/40 (12%).

**TABLE 1:** Demographics of the study population, at baseline.

Characteristics, at baseline	All (*n* = 40)
Age, years, mean ± SD	54 ± 14
Sex assigned at birth, *no*. (%)	
Female	33/40 (83)
Male	7/40 (18)
Menopausal, *no*. (%)	
Premenopausal	8/33 (24)
Perimenopausal	2/33 (6)
Postmenopausal	23/33 (70)
HADS score, *no*. (%)	
HADS‐A, median (IQR)	9 (7–11)
*Score 1–7*	13/35 (37)
*Score 8–10*	9/35 (26)
*Score 11–21*	13/35 (37)
HADS‐D, median (IQR)	5 (3–7)
*Score 1–7*	24/35 (69)
*Score 8–10*	7/35 (20)
*Score 11–21*	4/35 (11)

*Note:* Complete‐case initiator primary cohort (*n* = 40), unless indicated otherwise. Percentages may not sum to 100 because of rounding; counts are exact.

**TABLE 2:** Pain and clinical characteristics of the study population, at baseline.

Characteristics, at baseline	All (*n* = 40)
Pain duration, months, median (IQR)	24 (11–48)
Pain intensity, 0–10 NRS, mean ± SD	6.3 ± 1.7
Neuropathic screening	
DN4, 0–10, median (IQR)	3 (2–5)
DN4, ≥ 4 score, *no*. (%)	16 (40)
Injured trigeminal branch, *no*. (%)	
Maxillary (V2)	18/40 (45)
Mandibular (V3)	20/40 (50)
Both (V2 + V3)	2/40 (5)
Pain location, *no*. (%)	
Unilateral	35/40 (88)
Bilateral	5/40 (12)
Initiating event, *no*. (%)	
Tooth extraction	14/40 (35)
*Third molar*	6/40 (15)
*First/s molar*	6/40 (15)
*Premolar*	2/40 (5)
Root canal treatment	13/40 (33)
Dental implant placement	5/40 (13)
Periodontal surgery	4/40 (10)
Head trauma	2/40 (5)
Maxillofacial surgery	2/40 (5)
Prior systemic treatment failure^a^, no. (%)	40/40 (100)

*Note:* Complete‐case initiator primary cohort (*n* = 40).

^a^ Systemic treatment failure defined as prior use of an antidepressant, antiepileptic, or systemic analgesic with insufficient pain relief and/or intolerable adverse effects, leading to discontinuation (per medical record).

### 3.2 Primary Outcome

**FIGURE 1:**
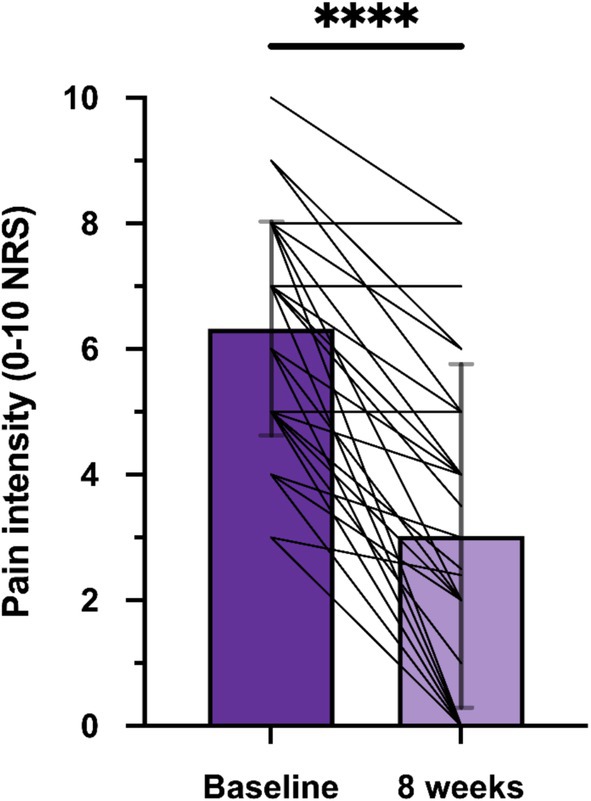
Individual and mean pain intensity scores at baseline and week 8. Each point represents an individual patient (*n* = 40). Lines connect paired measurements for the same patient. Mean ± SD are shown as horizontal lines and error bars. Mean pain intensity decreased from 6.3 ± 1.7 at baseline to 3.0 ± 2.7 at week 8, corresponding to a mean paired change of 3.30 NRS points (95% CI 2.51–4.09; paired *t*–test *p* < 0.001). Pain intensity was assessed using a 0–10 Numerical Rating Scale (NRS), where 0 indicates no pain and 10 indicates worst imaginable pain. A 2‐tailed *p* < 0.05 was considered significant. ****p < 0.001.

Mean pain intensity on the 0–10 NRS decreased from 6.3 ± 1.7 at baseline to 3.0 ± 2.7 at week 8. The mean within‐patient change from baseline to week 8 was 3.30 points (95% CI 2.51–4.09; t(39) = 8.46, *p* < 0.001), corresponding to a large effect size (Cohen's d_z_ = 1.34) (Table 3; Figure 1). A supportive nonparametric analysis showed concordant results, with a Hodges–Lehmann pseudo‐median paired difference of 3.25 points (95% CI 2.30–4.00; Wilcoxon signed‐rank *p* < 0.001).

**TABLE 3:** Primary and secondary outcomes, at week 8.

Pain characteristics, at week 8	All (*n* = 40)	(95% CI)
Pain intensity, 0–10 NRS, mean ± SD	3.0 ± 2.7	
Primary outcome–NRS change from baseline		
ΔNRS, mean (95% CI)	3.30	(2.51–4.09)
Secondary outcomes		
Responders rate, *no*. % (95% CI)		
*Patients with ≥ 50% reduction in pain intensity*	22/40	55 (40–69)
*Patients with ≥ 30% reduction in pain intensity*	27/40	68 (52–80)
Rating of global improvement, *no*. % (95% CI)		
*Patients with PGI‐I score 1–2* “*very much improved*” or “*much improved*”	30/40	75 (60–86)

*Note:* Primary results are presented as mean paired change with 95% CI and paired *t*–test *p*‐values. Supportive nonparametric analyses using the Wilcoxon signed‐rank test and Hodges–Lehmann pseudo‐median paired differences with 95% CIs showed concordant results. Unless otherwise noted, ΔNRS = baseline − week 8 (positive values indicate improvement). Complete‐case initiator primary cohort (*n* = 40).

### 3.3 Secondary Outcomes

At week 8, 22/40 patients (55%; 95% CI 40–69) achieved a ≥ 50% reduction in pain intensity and 27/40 patients (68%; 95% CI 52–80) achieved a ≥ 30% pain reduction. On the PGI‐I, 30/40 patients (75%; 95% CI 60–86) reported global improvement (score 1–2) (Table 3).

### 3.4 Safety

TEAEs occurred in 5/40 patients (12%; 95% CI 5–26); all were CTCAE grade 1, corresponding to asymptomatic or mild symptoms with no intervention indicated. Local AEs occured in 3/40 (7%; 95% CI 3–20), including oral dysesthesia in 2/40 (5%; 95% CI 1–16) and dysgeusia in 1/40 (2%; 95% CI 0–13). Systemic AEs occured in 2/40 patients (5%; 95% CI 1–16), including dizziness in 1/40 (2%; 95% CI 0–13) and fatigue in 1/40 (2%; 95% CI 0–13). No serious AEs (0%; 95% CI 0–9) or discontinuations due to AEs (0%; 95% CI 0–9) were reported. An additional clinical observation, not classified as treatment‐related, was removable white tongue coating in 1/40 (2%; 95% CI 0–13) (Table 4).

**TABLE 4:** Measure of adverse events (AEs).

Measure of adverse events (AEs)	All (*n* = 40)	(95% CI)
Treatment‐emergent AEs, *no. % (95% CI)*	5/40	12 (5–26)
Grade 1–asymptomatic or mild AEs	5/40	12 (5–26)
Local AEs	3/40	7 (3–20)
*Oral dysesthesia*	2/40	5 (1–16)
*Dysgeusia*	1/40	2 (0–13)
Systemic AEs	2/40	5 (1–16)
*Dizziness*	1/40	2 (0–13)
*Fatigue*	1/40	2 (0–13)
Serious AEs, *no. % (95% CI)*	0/40	0 (0–9)
AEs leading to discontinuations, *no. % (95% CI)*	0/40	0 (0–9)

*Note:* Treatment‐emergent adverse events (TEAEs), serious adverse events (SAEs), and adverse events (AEs) leading to discontinuations were reported, as graded by the Common Terminology Criteria for Adverse Events (CTCAE) v5.0.

### 3.5 Sensitivity Analyses

Estimates were directionally consistent across prespecified and post hoc sensitivity analyses, with similar magnitude (absolute differences in ΔNRS ranging from 0.00 to 0.39 points) and overlapping CIs relative to the primary analysis (Table S1). Differences in standardized response mean (Cohen's d_z_) largely reflected differences in the variance of change scores as well as mean change. Supportive nonparametric analyses using the Wilcoxon signed‐rank test and Hodges–Lehmann (HL) pseudo‐median paired differences were concordant across all sensitivity analyses.

(i) In the per‐protocol analysis restricted to participants adherent at week 8 (*n* = 36): mean change was 3.67 (95% CI 2.88–4.46; paired *t*–test *p* < 0.001). The HL pseudo‐median paired difference was 3.50 (95% CI 2.30–4.00; Wilcoxon signed‐rank *p* < 0.001).

(ii) After exclusion of patients with systemic analgesic changes during follow‐up: no patients had initiated or changed systemic analgesics; estimates were therefore identical to the primary analysis.

(iii) After exclusion of patients receiving concurrent systemic tricyclics at baseline (*n* = 39): mean change was 3.39 (95% CI 2.60–4.18; paired *t*–test *p* < 0.001). The HL pseudo‐median paired difference was 3.50 (95% CI 2.50–4.00; Wilcoxon signed‐rank *p* < 0.001).

(iv) In baseline pain strata of NRS < 6 (*n* = 16) and NRS ≥ 6 (*n* = 24): mean changes were 2.91 (95% CI 2.04–3.78) and 3.56 (95% CI 2.34–4.79), respectively; paired *t*–test *p* < 0.001 in both strata. The HL pseudo‐median paired differences were 3.00 (95% CI 2.00–3.75) and 3.50 (95% CI 1.75–4.25), respectively; Wilcoxon signed‐rank *p* < 0.001 in both strata.

(v) In analyses restricted to patients with pain duration ≥ 3 months (*n* = 38) and ≥ 6 months (*n* = 33): mean changes were 3.29 (95% CI 2.46–4.12) and 3.25 (95% CI 2.34–4.15), respectively; paired *t*–test *p* < 0.001 in both analyses. The HL pseudo‐median paired differences were 3.25 (95% CI 2.25–4.00) and 3.00 (95% CI 1.50–3.50), respectively; Wilcoxon signed‐rank *p* < 0.001 in both analyses.

(vi) In post hoc pain‐duration strata of < 12 months (*n* = 11) and ≥ 12 months (*n* = 29): mean changes were 3.15 (95% CI 1.81–4.48) and 3.36 (95% CI 2.35–4.37), respectively; paired *t*–test *p* < 0.001 in both strata. The HL pseudo‐median paired differences were 3.00 (95% CI 1.12–3.50) and 3.25 (95% CI 2.00–4.00), respectively; Wilcoxon signed‐rank *p* < 0.001 in both strata.

## 4 Discussion

To our knowledge, this retrospective real‐world evidence study is the first to investigate the effectiveness and tolerability of topical amitriptyline for PTNP. In 40 adults with PTNP, an intraoral mucoadhesive gel containing amitriptyline 10% plus lidocaine 2% was associated with a substantial within‐patient reduction in pain intensity at 8 weeks, with more than half of patients achieving a ≥ 50% reduction in pain intensity and few reported adverse events. Although causal inference is limited by the single‐arm design, the observed mean 3.30‐point reduction in NRS pain intensity is clinically important, exceeds established IMMPACT thresholds for clinically meaningful improvement in chronic pain, and remained consistent across prespecified sensitivity analyses.

### 4.1 Clinical Context and Interpretation of Treatment Response

These findings should be interpreted in the context of the limited effectiveness and tolerability of currently used systemic treatments for PTNP in routine care. Real‐world data suggest that most patients with PTNP experience persistent symptoms despite stepped pharmacotherapy, often with burdensome adverse effects and unsatisfactory longer‐term outcomes [13, 46, 47]. For example, Haviv et al. reported that only 11% of patients with PTNP achieved a ≥ 50% reduction in pain intensity after stepped systemic pharmacotherapy including tricyclic antidepressants, anticonvulsants, and combination regimens [13]. Likewise, Liu et al. found that pregabalin monotherapy provided satisfactory pain relief in only 28% of patients with PTNP following trigeminal neuralgia‐related neuroablative procedures, with systemic adverse events reported in 97% of patients [47]. Against this background, the responder rates observed in the present study (i.e., 55% for ≥ 50% pain reduction and 68% for ≥ 30% pain reduction) appear favourable relative to what is typically reported for systemic neuropathic pain therapies [11] and align with IMMPACT recommendations for substantial and moderate improvement, respectively [45]. In addition, 75% of patients reported improvement on the PGI‐I (scores 1–2), further suggesting that the reduction in pain intensity was accompanied by a clinically meaningful patient‐perceived benefit. Taken together, these observations support the potential practical relevance of topical treatment strategies in PTNP, particularly when standard systemic options are ineffective, poorly tolerated, or both.

At the same time, the relatively high response rates observed here should be interpreted cautiously. They may reflect, in part, route‐specific therapeutic advantages of local mucosal delivery, as discussed below, but they may also have been amplified by the retrospective single‐arm design, regression to the mean, contextual effects, and selection of treatment initiators with available follow‐up. The present results therefore support a promising clinical signal rather than definitive comparative efficacy.

### 4.2 Route‐ and Formulation‐Specific Therapeutic Rationale

In this study, the magnitude of improvement observed with intraoral delivery may reflect route‐specific pharmacological advantages of mucosal administration over systemic or dermal routes. The oral mucosa demonstrates approximately 10‐fold greater drug permeability than intact skin, is densely innervated by trigeminal afferents, and provides direct access to superficial peripheral nerve terminals implicated in PTNP pathophysiology [19, 20, 21]. This anatomical configuration may allow therapeutically relevant local drug exposure at the painful site while limiting the systemic burden typically associated with oral TCAs.

This interpretation is also relevant to formulation choice. Much of the topical neuropathic pain literature has focused on dermal preparations containing ketamine, pregabalin, baclofen, or higher‐concentration lidocaine, whereas the evidence base for topical amitriptyline has been more heterogeneous [14, 23, 24, 25, 27, 48]. In skin‐based studies, concentrations of ≤ 5% have generally shown limited or inconsistent benefit, whereas higher concentrations, often around 10% or more, have shown more encouraging signals [14, 23, 24, 25, 27, 48]. Formulation and permeation data further suggest poor permeation from common bases below 5% and improved flux at ≥ 5% [49], supporting the concept that the performance of topical amitriptyline depends not only on nominal concentration but also on vehicle characteristics and the biological barrier crossed.

Conversely, the mucosal and orofacial literature suggests that intraoral delivery of topical tricyclic formulations may produce rapid, potent, long‐lasting, and well‐tolerated analgesic effects across nociceptive, neuropathic, and nociplastic trigeminal pain conditions. In healthy volunteers, intraoral 10 mg amitriptyline mucoadhesive tablets induced complete mucosal anaesthesia in a split‐mouth randomized controlled study [31], and a comparative randomized clinical trial of 2% intraoral amitriptyline versus 2% lidocaine suggested that amitriptyline can exert relevant local anaesthetic and analgesic effects [30]. In radiochemotherapy‐induced oral mucositis, TCAs are commonly used in mouthwash preparations for pain management and have shown meaningful short‐term analgesic benefit with high‐level evidence [36, 37]. A comparative randomized clinical trial showed greater short‐term pain reduction with 0.1% amitriptyline oral rinses than with benzydamine, with peak reductions of 95%–99% in pain intensity over the first 10–30 min [33]. Alongside amitriptyline, the closely related TCA doxepin, used as a 0.5% mouthrinse, showed substantial pain reduction within 5–15 min and sustained relief for 4 h [34, 35, 38]. In acute inflammatory dental pain, intrapulpal 2% amitriptyline decreased pain intensity by 93% within 9 min despite prior lidocaine failure, reinforcing its potential for additional potent local sodium‐channel blockade at trigeminal endings [39]. Topical amitriptyline has likewise gained interest in burning mouth syndrome [50], a debilitating nociplastic disorder with trigeminal small‐fibre neuropathy characteristics [42]. A recent randomized clinical trial using 0.01%–0.025% amitriptyline mouthwash showed significant dose‐responsive efficacy and global improvement [32], aligning with findings from our prior real‐world evidence study using equivalent 0.025% dosing [29], thus supporting the biological plausibility of this approach in PTNP.

Additional support for a broader “route matters” hypothesis comes from other highly permeable mucosal pain settings. Low‐concentration amitriptyline formulations of 0.5%–2% have been associated with substantial clinical improvement (ranging from 64% to 85%) in vulvodynia, dyspareunia, and pudendal neuralgia studies [51, 52, 53, 54], as summarized in recent state‐of‐science reviews [55]. Taken together, these data support a concentration–route hypothesis: skin‐based amitriptyline efficacy appears more likely to emerge above a practical release/permeation threshold in the 5%–10% range, whereas mucosal delivery across a more permeable barrier and onto nociceptor‐dense tissue may achieve meaningful analgesia at lower nominal concentrations. This framework helps reconcile negative low‐dose RCTs, mixed dermal findings at higher concentrations, and the stronger signals reported with mucosal amitriptyline in experimental and clinical settings, while acknowledging the limitations of cross‐trial comparisons. This concentration–route perspective is consistent with the improvement observed in the present cohort and with prior reports of topical tricyclic analgesia across mucosal pain conditions.

### 4.3 Mechanistic Plausibility

The analgesic effect of intraoral topical amitriptyline is biologically plausible, although the underlying mechanism cannot be established from the present study. In contrast to orally administered amitriptyline, whose effects are usually framed in terms of central monoamine reuptake inhibition, topical amitriptyline is more likely to act predominantly through peripheral membrane and ion‐channel effects [25, 28, 48, 56]. The strongest mechanistic support concerns local blockade of voltage‐gated sodium channels. Experimental studies indicate that amitriptyline can function as a potent and relatively long‐acting local anaesthetic, including effects on nociceptor‐associated sodium channels such as Na_v_1.7, Na_v_1.8 and Na_v_1.9, thereby reducing ectopic activity and peripheral sensitization after nerve injury [56, 57]. Preclinical studies further suggest that amitriptyline may exert more potent local anaesthetic effects than several other TCAs and bupivacaine and, in some experimental models, may show a more favourable differential block with relatively greater nociceptive than motor blockade [56, 57]. Lidocaine may complement this profile through more rapid sodium‐channel blockade, potentially contributing to early local analgesia while amitriptyline sustains the effect.

Beyond sodium‐channel blockade, additional peripheral mechanisms may also be involved, although support for these pathways remains more inferential. TRPV1 and TRPA1 are major canonical nociceptor gates, acting as key integrators of capsaicin, heat, and protons, and electrophiles, irritants, and cold, respectively; they are co‐expressed and cross‐sensitized in trigeminal nociceptive pathways and contribute to irritant signalling, neurogenic inflammation, and peripheral sensitization [58, 59, 60]. Notably, preclinical trigeminal models suggest that TRPV1 activation can drive TRPA1‐dependent sensitization in trigeminal nociceptive pathways [61], and that TRPA1 antagonism can reverse allodynia while downregulating Trpa1/Trpv1 expression and pro‐inflammatory mediators, implicating a TRPA1–TRPV1 neuroimmune loop in trigeminal neuropathic pain [62]. High local concentrations of amitriptyline can also activate and then desensitize TRPV1, while lidocaine may transiently gate and subsequently inhibit or desensitize TRPV1/TRPA1 [63, 64]. By modulating TRPV1/TRPA1 signalling, the amitriptyline–lidocaine combination may have limited irritant input and contributed to local tolerability despite the high nominal amitriptyline concentration. TRPV1 opening can also facilitate intracellular access and retention of cationic local anaesthetics, deepening Na_v_ blockade at the terminal, as shown for amitriptyline in human skin and supported by voltage‐dependent gating studies [22, 65]. Amitriptyline has also been reported to exert state‐dependent NMDA (N‐methyl‐D‐aspartate) receptor actions, enhancing Ca^2+^‐dependent desensitization at low micromolar concentrations and producing open‐channel trapping block at higher levels [66]. In this context, such effects could further dampen glutamatergic sensitization and abnormal excitability at injured terminals, acting as a complementary inhibitory mechanism, although this remains speculative in the present context.

Whether these channel‐level actions occur at clinically relevant concentrations after repeated intraoral application in humans remains uncertain. In addition, some systemic absorption cannot be excluded. Because the oral mucosa is highly permeable and vascularized, a fraction of the applied dose may have entered the systemic circulation and contributed to analgesia [19, 21]. However, the intended local mode of delivery, the low frequency of reported systemic adverse events in the present cohort, and the rapid analgesic effects described with intraoral tricyclic formulations in prior studies are collectively more consistent with a predominantly peripheral mechanism than with a delayed centrally mediated effect. Overall, the present findings support mechanistic plausibility rather than mechanistic proof, and formal pharmacokinetic and mechanistic studies will be needed to clarify the relative contribution of local and systemic effects.

### 4.4 Safety Profile

The favourable safety profile represents a potentially important clinical advantage. Only 5 of 40 patients (12%) reported adverse events; all were mild, and most were local, with no serious adverse events and no treatment discontinuations. This profile compares favourably with systemic tricyclic therapy, in which anticholinergic effects, sedation, dry mouth, constipation, cardiovascular concerns, and cognitive impairment often limit dosing and long‐term adherence in clinical practice [11, 12]. The present safety profile is also consistent with prior studies of high‐concentration topical amitriptyline in chemotherapy‐induced peripheral neuropathy, which likewise did not identify clear systemic adverse events [23, 24]. This pattern may reflect the advantages of targeted local delivery, which could permit therapeutically relevant mucosal exposure while limiting systemic burden. However, pharmacokinetic studies and ECG monitoring are needed to better define systemic exposure and cardiac safety with repeated intraoral use.

### 4.5 Limitations

Beyond its strengths, this study has several important limitations. First, the single‐arm observational design inherently limits causal inference. Changes in pain intensity and global improvement cannot be attributed solely to the intervention, because natural recovery, regression to the mean, increased disease awareness, behavioural counselling and concurrent changes in lifestyle and psychosocial factors, including stress, sleep, diet, physical activity, and social functioning, may also have contributed. Second, the single‐centre tertiary care setting may limit generalisability, as the study population may represent more severe or treatment‐refractory cases than patients managed in community settings. Third, we did not characterize the early time course of analgesia over the first few hours after intraoral application, limiting pharmacodynamic insight and comparison with cutaneous topical studies. In addition, because retention time was not measured directly and no stent‐based delivery system was used, we could not determine the relative contributions of mucoadhesion and the standardized brief contact‐time protocol to the observed clinical response. Fourth, the effectiveness analysis was restricted to treatment initiators; although appropriate for estimating on‐treatment change, this approach may overestimate benefit relative to an intention‐to‐treat analysis. Fifth, because the formulation combined amitriptyline with lidocaine, the independent contribution of amitriptyline cannot be determined, and potential additive or synergistic effects cannot be excluded. Finally, the absence of standardized quantitative sensory testing limited characterization of the somatosensory phenotype and exploration of whether treatment response differed according to baseline sensory dysfunction.

### 4.6 Clinical Implications and Future Directions

These findings suggest that intraoral topical amitriptyline–lidocaine could represent a practical local treatment option for patients with PTNP and may help address an important unmet therapeutic need in a population often exposed to sequential or combined systemic treatment regimens. As a medication‐sparing approach, it may be particularly relevant when systemic therapies are ineffective, poorly tolerated, or difficult to sustain [67]. From a practical standpoint, the intraoral gel formulation is attractive because it is easy to apply, can be self‐administered, and integrates readily into routine dental and oral rehabilitation care. More broadly, the present results support further development of mechanism‐based topical and drug‐repurposing strategies for trigeminal neuropathic pain conditions in which local peripheral mechanisms are clinically important.

Future work should now focus on prospective controlled validation. A prospectively registered, randomized, controlled trial with prespecified outcomes and repeated follow‐up should confirm these findings, assess dose–response relationships, and determine the role of topical amitriptyline in PTNP treatment pathways. Pharmacokinetic assessment and ECG monitoring will be important to define systemic exposure and cardiac safety with repeated intraoral use. Further optimisation of formulation parameters, including mucoadhesion, palatability, concentration, contact time, and dosing frequency, together with sensory phenotyping, may help refine patient selection and improve clinical utility.

## 5 Conclusions

In this retrospective real‐world study, intraoral amitriptyline 10% plus lidocaine 2% gel was associated with substantial pain improvement and favourable tolerability in adults with PTNP. These findings support targeted mucosal delivery as a promising local therapeutic option and a potential medication‐sparing strategy, particularly when systemic therapies are insufficient or poorly tolerated. More broadly, they provide a rationale for further investigation of this approach in other mucosal trigeminal pain settings. Randomised controlled trials are now needed to confirm efficacy, optimise formulation and dosing parameters, and clarify the role of topical amitriptyline in PTNP treatment pathways and routine clinical care.

## Author Contributions

A.L. had full access to all the data in the study and takes responsibility for the integrity of the data and the accuracy of the data analysis. A.L.: conceptualization; data curation; formal analysis; investigation; methodology; project administration; resources; software; supervision; validation; visualization; writing – original draft; writing – review and editing. I.S.: data curation; formal analysis; investigation; writing – review and editing. Y.B.: data curation; investigation; methodology; project administration; resources; supervision; validation; visualization; writing – review and editing.

## Disclosure

The authors are aware of patent families assigned to Algotherapeutix and Laboratoires Mayoly Spindler relating to topical amitriptyline formulations and their therapeutic use in neuropathic pain, including FR3065371B1 and FR3108841B1 and corresponding family members. The product evaluated in the present study was an extemporaneous, unit‐dose magistral preparation compounded in a pharmacy on a named‐patient prescription, as described in the Methods; such preparations are addressed by Article L613‐5(c) of the French Intellectual Property Code. The authors do not own, claim, or seek intellectual property rights related to the formulation, compounding process, or therapeutic use evaluated in this study.

## Ethics Statement

The study complied with the Declaration of Helsinki and French data‐protection regulations (National Data Protection Agency registration #DR‐2020‐341). The Assistance Publique‐Hôpitaux de Paris Institutional Review Board approved this study (INDS‐TPS#1106180) and waived informed consent owing to retrospective, de‐identified data collected in routine clinical care.

## Consent

The authors have nothing to report.

## Conflicts of Interest

The authors declare no conflicts of interest.

## Supporting information


**Figure S1:** Cohort flow diagram and analysis sets.

**Table S1:** Prespecified and post hoc sensitivity analyses of within‐patient change in NRS pain intensity from baseline to week 8.

**Table S2:** Time from injury to treatment, stratified by PTNP aetiology.

## Data Availability

The data that support this study's findings are available from AP‐HP, but restrictions apply. These data were used under license for the current research and are not publicly available. However, data are available from the authors upon reasonable request and with permission of the AP‐HP institutional review board and Sorbonne University ethics committee.
