# Supplementary material for: Effectiveness and Tolerability of Topical Amitriptyline 10% Plus Lidocaine 2% Gel in Adults With Post‐Traumatic Trigeminal Neuropathic Pain: A Real‐World Evidence Study
Source: J Oral Rehabil. 2026 May 5;53(8):1550–61. doi: 10.1111/joor.70209 (PMC13358445; doi:10.1111/joor.70209)
Supplement: Supplementary file 1 — Figure S1: Cohort flow diagram and analysis sets. [file JOOR-53-1550-s002.docx]

## **Figure S1. Cohort flow diagram and analysis sets**

Flow of 94 patients screened from electronic medical records to the primary complete-case cohort (n = 40) and prespecified sensitivity subsets. Boxes indicate patient counts at each stage: eligibility assessment (n = 48), baseline NRS available, week-8 NRS available, and exclusions (with reasons and numbers). Arrows denote study progression. Sensitivity sets include per-protocol (n = 36), stable medication (n = 40), no concurrent TCAs (n = 39), baseline NRS <6 (n = 16), baseline NRS ≥6 (n = 24), duration ≥3 months (n = 38), and duration ≥6 months (n = 33).
