# Supplementary material for: Effectiveness and Tolerability of Topical Amitriptyline 10% Plus Lidocaine 2% Gel in Adults With Post‐Traumatic Trigeminal Neuropathic Pain: A Real‐World Evidence Study
Source: J Oral Rehabil. 2026 May 5;53(8):1550–61. doi: 10.1111/joor.70209 (PMC13358445; doi:10.1111/joor.70209)
Supplement: Supplementary file 2 — Table S1: Prespecified and post hoc sensitivity analyses of within‐patient change in NRS pain intensity from baseline to week 8. [file JOOR-53-1550-s003.docx]

Table S1. Prespecified and post hoc sensitivity analyses of within-patient change in NRS pain intensity from baseline to week 8

| **Sensitivity analyses^#^** | n | Mean change  (95% CI) | HL pseudo-median change  (BCa 95% CI)^¶^ | *p* | SRM | Difference in ΔNRS compared to  primary analysis |
| --- | --- | --- | --- | --- | --- | --- |
| (i) Per-protocol (adherent at week 8) | 36 | 3.67 (2.88–4.46) | 3.50 (2.30–4.00) | <0.0001 | 1.58 | + 0.37 |
| (ii) Stable medication regimen during 8 weeks | 40 | 3.30 (2.51–4.09) | 3.25 (2.30–4.00) | <0.0001 | 1.34 | + 0.00 |
| (iii) No concurrent systemic TCAs at baseline | 39 | 3.39 (2.60–4.18) | 3.50 (2.50–4.00) | <0.0001 | 1.39 | + 0.09 |
| (iv) Baseline pain strata, at baseline |  |  |  |  |  |  |
| NRS <6 | 16 | 2.91 (2.04–3.78) | 3.00 (2.00–3.75) | <0.0001 | 1.78 | - 0.39 |
| NRS ≥6 | 24 | 3.56 (2.34–4.79) | 3.50 (1.75–4.25) | <0.0001 | 1.23 | + 0.26 |
| (v) Pain duration- restricted, at baseline |  |  |  |  |  |  |
| ≥3 months | 38 | 3.29 (2.46–4.12) | 3.25 (2.25–4.00) | <0.0001 | 1.30 | - 0.01 |
| ≥6 months | 33 | 3.25 (2.34–4.15) | 3.00 (1.50–3.50) | <0.0001 | 1.27 | - 0.05 |
| (vi) Pain duration- restricted, at baseline |  |  |  |  |  |  |
| <12 months | 11 | 3.15 (1.81–4.48) | 3.00 (1.12–3.50) | <0.0001 | 1.58 | - 0.15 |
| ≥12 months | 29 | 3.36 (2.35–4.37) | 3.25 (2.00–4.00) | <0.0001 | 1.26 | + 0.06 |

Sensitivity analyses assess the robustness of the primary complete-case analysis (n = 40), for which the mean paired change was 3.30 points (95% CI 2.51–4.09; paired t test p < 0.001). Mean change with 95% CI is shown as the primary summary. Supportive nonparametric analyses used the Wilcoxon signed-rank test and Hodges–Lehmann pseudo-median paired differences with 95% CIs. ^#^All sensitivity analyses were prespecified except analysis (vi), which was post hoc. ΔNRS = baseline − week 8. ^¶^Hodges–Lehmann 95% CIs were obtained using bias-corrected and accelerated bootstrap with 10,000 paired resamples. Abbreviations: BCa = bias-corrected accelerated; CI = confidence interval; HL = Hodges-Lehmann; NRS = numeric rating scale; SRM = standardized response mean; TCA = tricyclic antidepressant.
