# Supplementary material for: Effectiveness and Tolerability of Topical Amitriptyline 10% Plus Lidocaine 2% Gel in Adults With Post‐Traumatic Trigeminal Neuropathic Pain: A Real‐World Evidence Study
Source: J Oral Rehabil. 2026 May 5;53(8):1550–61. doi: 10.1111/joor.70209 (PMC13358445; doi:10.1111/joor.70209)
Supplement: Supplementary file 3 — Table S2: Time from injury to treatment, stratified by PTNP aetiology. [file JOOR-53-1550-s001.docx]

## **Table S2. Time from injury to treatment, stratified by PTNP etiology**

| **Characteristics** | All (n = 40) | P value |
| --- | --- | --- |
| Time from injury to treatment, months, median (IQR) | 24 (11–48) |  |
| Tooth extraction | 24 (8–39) | \| 0.957 \| \| --- \| |
| Root canal treatment | 24 (18–54) |  |
| Dental implant placement | 33 (3–210) |  |
| Periodontal surgery | 27 (13–45) |  |
| Head trauma | 43 (3–84) |  |
| Maxillofacial surgery | 30 (13–48) |  |

Complete-case initiator primary cohort (n = 40) included tooth extraction 14/40 (35%), root canal treatment 13/40 (33%), dental implant placement 5/40 (13%), periodontal surgery 4/40 (10%), head trauma 2/40 (5%), and maxillofacial surgery 2/40 (5%). Time from injury to treatment (months) is reported as median (IQR) overall and by PTNP etiology. Groups were compared using the Kruskal–Wallis test (6 groups; total n=40; approximate P=0.957; H=1.067), providing no evidence of differences across etiologic categories. Group sizes were uneven (including two categories with n=2), consequently statistical power to detect differences was limited.
